# PCRX-201, a novel IL-1Ra gene therapy treatment approach for low back pain resulting from intervertebral disc degeneration

**DOI:** 10.1038/s41434-024-00504-7

**Published:** 2024-11-21

**Authors:** Joseph W. Snuggs, Rebecca K. Senter, Joshua P. Whitt, J. Derek Jackson, Christine L. Le Maitre

**Affiliations:** 1 Division of Clinical Medicine, The University of Sheffield, Sheffield, UK; 2 Biomolecular Sciences Research Centre, Sheffield Hallam University, Sheffield, UK; 3 Former employee of Flexion Therapeutics, a wholly owned subsidiary of Pacira Biosciences, Tampa, Florida USA; 4 Pacira Pharmaceuticals Inc, Tampa, Florida USA

**Keywords:** Cell biology, Rheumatic diseases

## Abstract

Low back pain is the leading cause of global disability with intervertebral disc (IVD) degeneration a major cause. However, no current treatments target the underlying pathophysiological causes. PCRX-201 presents a novel gene therapy approach that addresses this issue. PCRX-201 codes for interleukin-1 receptor antagonist, the signalling inhibitor of the pro-inflammatory cytokine interleukin-1, which orchestrates the catabolic degeneration of the IVD. Here, the ability of PCRX-201 to transduce human nucleus pulposus cells to increase IL-1Ra production was assessed together with effects on catabolic pathways. When transduced with PCRX-201, the production and release of IL-1Ra was increased in degenerate human nucleus pulposus cells and tissue. Whereas, the production of downstream proteins, including IL-1β, IL-6, MMP3, ADAMTS4 and VEGF were decreased in both cells and tissue, indicating a reduction in IL-1-induced catabolic signalling. Here, a novel gene therapy vector, PCRX-201, was shown to transduce degenerate NP cells and tissue, increasing the production of IL-1Ra. The increased IL-1Ra resulted in decreased production of catabolic cytokines, enzymes and angiogenic factors, whilst also increasing aggrecan expression. This demonstrates PCRX-201 enables the inhibition of IL-1-driven IVD degeneration. The ability of PCRX-201 to elicit anti-catabolic responses is promising and warrants further development to determine the efficacy of this exciting, novel gene therapy.

## Introduction

Low back pain (LBP) is the leading cause of disability worldwide [1]. Almost half of chronic LBP cases are attributable to degeneration of the intervertebral disc (IVD) [2–4]. Patients suffering from chronic LBP due to IVD degeneration who have exhausted conservative treatments, such as pain relief medication and physiotherapy, have no remaining options other than invasive and costly surgical intervention, which are only applicable to a small sub-set of patients [5]. To date, there are no treatments that halt or reverse the biological causes of IVD degeneration [5], despite its profound socioeconomic burden and impact, decreasing the quality of life for millions of people [1].

The IVD is a unique structure, composed of three distinct tissues: the nucleus pulposus (NP), annulus fibrosus (AF), and cartilaginous endplate (CEP). All three work in unison to enable the function of the IVD; to withstand compressive forces and enable movement of the spine [6]. The function of the native IVD cells is governed by multiple environmental cues such as biomechanics, fluctuating osmotic pressure, minimal nutrient diffusion, and low oxygen tension, which are all finely balanced [7, 8]. If this is disrupted, the balance can be shifted towards catabolism and degeneration of the tissue.

IVD degeneration is characterised by progressive changes to the extracellular matrix (ECM) due to altered cellular metabolism, matrix synthesis, increased degradation of normal matrix components, and changes in the composition of the ECM [9–11]. Matrix degradation is accelerated by the upregulation of degradative enzymes, including MMPs (matrix-metalloproteinases) and ADAMTS (a-disintegrin-and-metalloproteinase-with-thrombospondin-motifs) [12, 13]. Compositional changes in the matrix during IVD degeneration are accompanied by cellular changes, including apoptosis and senescence [14–16], leading to decreased tissue cellularity [17] and contribute to increased secretion of senescence associated secretory factors such as MMPs[14]. Nerve and blood vessel ingrowth into the disc occurs, which may lead to sensitization and pain [18–20]. IVD cells produce a plethora of catabolic cytokines and chemokines [17, 21–23] with highest expression seen in the NP and inner AF [21, 23, 24].

There is increasing evidence supporting the role of a pivotal cytokine in the pathogenesis of IVD degeneration: interleukin (IL)-1. The levels of IL-1 expression increase with IVD degeneration, which in turn increases the expression of degradative enzymes, and decreases the expression of ECM genes [24], leading to the destruction of tissue. Interestingly, the expression of the natural inhibitor of IL-1 signalling, IL-1 receptor antagonist (IL-1Ra), is not increased during degeneration [24], indicating IL-1 signalling becomes unbalanced, and inhibition of IL-1 in vitro has also been shown to completely abrogate matrix degradation [25]. However, the presence IL-1 is also essential for IVD health [26], indicating this cytokine has an important role in IVD homeostasis, which is disrupted during degeneration. The importance of IL-1 was further defined by the observation that spontaneous degeneration occurred when IL-1Ra was knocked out in mouse models [27, 28], which is supported by the findings that polymorphisms in the IL-1 gene cluster increases the risk of IVD degeneration and LBP [29–31].

Thus, inhibiting the increased expression of IL-1 within the degenerate disc could be an attractive strategy to halt degeneration and enable tissue regeneration for the treatment of LBP. A potential strategy to achieve this would be to increase IL-1Ra within the disc. This has been investigated previously, where delivery of IL-1Ra, encapsulated in poly(lactic-co-glycolic acid) (PLGA) microspheres, enabled glycosaminoglycan (GAG) retention in vitro and in vivo [32, 33]. IVD cells have also been previously shown to be capable of gene transfer of IL-1Ra with an adenoviral vector [34]. However, only non-degenerate cells showed increased expression of IL-1Ra protein following gene transfer [35]. IL-1Ra levels were successfully increased in IVD tissues following injection of non-degenerate disc cells, but not degenerate IVD cells, infected with the IL-1Ra adenoviral vector [35]. Furthermore these discs also displayed decreased MMP production, demonstrating a gene therapy approach targeting the delivery of functional IL-1Ra is promising [34, 35].

IL-1Ra gene therapy also shows promise for the treatment of osteoarthritis, which has a similar disease aetiology as IVD degeneration [36]. Previous pre-clinical studies have shown that IL-1Ra gene therapy for OA has disease-modifying effects and encouraging safety profiles [37–39]. One such gene therapy is PCRX-201 (formerly called FX201), which is a helper-dependent serotype 5-based High-capacity adenoviral (HDAd) vector designed to produce IL-1Ra under the control of a NF-κB promoter [37]. PCRX-201 reduced structural changes and chondrocyte loss within an anterior cruciate ligament transection (ACLT) rat model of OA, effectively decreasing joint damage with no adverse effects, and a Phase 1 study (NCT04119687) to assess safety and tolerability in patients is currently underway [37].

Therefore, we hypothesise that the therapeutic effects of PCRX-201 on OA disease progression could be translated to IVD degeneration. Specifically, this study aimed to determine if PCRX-201 can induce IL-1Ra protein expression in human IVD cells derived from patients with degenerate discs, identify the maintenance of PCRX-201 and long-term release profile of IL-1Ra, and investigate the efficacy of IL-1Ra induction by PCRX-201 to decrease the production of catabolic features of IVD degeneration. Finally, the ability of PCRX-201 to induce IL-1Ra production in ex vivo human IVD tissue, via direct injection was investigated.

## Materials and methods

### Vector production & viral count determination

PCRX-201 was manufactured under Good Manufacturing Practices (GMPs) using an adhesion culture manufacturing system. The upstream process employed a modified HEK-293 cell-line expressing recombinase enzyme. Producer cells were co-infected with PCRX-201, a high-capacity helper-dependent adenoviral vector (HCAd) and a helper virus (HV). PCRX-201 was trans-propagated, borrowing elements encoded in the HV genome and the packaging cell. HV propagation was limited by recombinase excision of the HV packaging signal. Packaging cells were harvested, lysed, and the resulting lysate digested in an enzymatic system to remove fragmented DNA, and subsequently purified through multiple cycles of ultra-centrifugation. Purified PCRX-201 was then buffer exchanged by dialyzing into the final formulation buffer and diluted to the target concentration. Viral titre of PCRX-201 was determined as 5.8 × 10^12^ Viral Particles per millilitre (VP/mL) by UV Optical Density at 260 nm. Note whilst infectious titre was not specifically calculated for the batch utilised within this study, the genomic titre to infectivity ratio of 5 batches of representative material generated equivalently were 75.9 (mean) with a range of 29–168.

### Human IVD sample collection

Human NP tissue was collected (Table 1a–c), with informed consent, from patients undergoing microdiscectomy surgery for the treatment of nerve root compression following IVD herniation. Ethical approval was granted by Sheffield Research Ethics Committee (09/H1308/70 / IRAS 10266). Tissue was fixed for 48 h in 10% Neutral Buffered Formalin (3800600, Leica Biosystems, Milton Keynes, UK), embedded into wax and 4 µm sections were produced. Sections of human NP tissue were used to determine the histological grade of degeneration following previously published methods, utilising the criteria for grading NP tissue regions from 0 to 9 (0 to 3 non-degenerate, 4 to 6 mid-grade degenerate, 7 to 9 severely degenerate) [40].

**Table 1:** Patient information for samples used in during (a) monolayer culture, (b) 3D alginate bead culture and (c) tissue explant culture for viability testing, IL-1Ra and downstream protein production. Histological grade of degeneration: non-degenerate (0–3), mid-grade (4–6), severe (7–9). (d) Antibody dilutions and antigen retrieval methods used for immunohistochemistry experiments.

Patient no.	Patient ID	Sex	Age	IVD level	Grade of degeneration
***a. Monolayer viability and IL-1Ra production***					
1	523	F	74	L4/L5	6
2	534	M	29	L5/S1	5
3	546	F	21	L5/S1	8
4	559	M	50	L4/L5	5
5	560	M	51	L3/L4	5
6	563	M	44	L3/L4	7
7	569	M	53	L5/S1	8
***b. 3D long-term IL-1Ra production, viability, downstream protein production, paracrine effects***					
1	577	F	72	L3/L4	7
2	580	F	56	L4/L5	5
3	588	M	49	L5/S1	8
4	593	F	47	L4/L5	6
5	596	M	21	L4/L5	2
6	599	M	29	L4/L5	6
***c. Downstream protein production in human NP explant tissue***					
1	600	M	41	L5/S1	5
2	618	M	38	L4/L5	7
3	623	M	74	L4/L5	8
4	624	F	22	L5/S1	4
5	625	F	42	L5/S1	6
***d. Antibody dilutions and antigen retrieval used in immunohistochemistry***					

*Target*	*Clonality*	*Dilution*	*Antigen retrieval method*	*Secondary antibody*
IL-1Ra (ab124962)	Rabbit monoclonal	1in400	None	Goat anti rabbit (ab6720)
IL-1β (ab9722)	Rabbit polyclonal	1in100	Heat	Goat anti rabbit (ab6720)
VEGFA (ab52917)	Rabbit monoclonal	1in100	Enzyme	Goat anti rabbit (ab6720)
NGF (ab52918)	Rabbit monoclonal	1in100	Enzyme	Goat anti rabbit (ab6720)
Collagen type II (ab34712)	Rabbit polyclonal	1in200	Enzyme	Goat anti rabbit (ab6720)
Aggrecan (ab3778)	Mouse monoclonal	1in100	Heat	Rabbit anti mouse (ab6727)
MMP3 (ab53015)	Rabbit polyclonal	1in400	Enzyme	Goat anti rabbit (ab6720)
ADAMTS4 (ab185722)	Rabbit polyclonal	1in200	None	Goat anti rabbit (ab6720)

All antibodies supplied by Abcam (Cambridge, UK).

### Human NP cell isolation

NP cells were isolated from tissue by enzymatic digestion with 64 U/mL collagenase type II (Life-Technologies, Paisley, UK) for 4 hr at 37 °C with shaking, before filtering with a 70 µM cell strainer [41]. Isolated NP cells were expanded in monolayer culture up to passage 2 before experimental use. All cultures were tested for mycoplasma prior to experimental procedures and were mycoplasma negative throughout the study. Cell expansion was undertaken using DMEM + GlutaMAX™ (4.5 g/L D-Glucose, +Pyruvate) (31966-021, Life-Technologies) supplemented with 1% (v/v) Penicillin-Streptomycin (15070063, Life-Technologies), 2.5 µg/mL amphotericin B solution (A2942, Sigma-Aldrich, Gillingham, UK), 50 µg/mL L-Ascorbic acid 2-phosphate sesquimagnesium salt hydrate (A8960, Sigma-Aldrich) and 10% (v/v) foetal bovine serum (Life-Technologies) [41].

### Human NP tissue explant isolation

To extract ex vivo NP tissue explants, surrounding tissues were removed and NP tissue dissected into 5 mm^3^ explants. Explants were each placed into individual plastic rings (⌀ 5mm), to semi-constrain and limit tissue swelling, and cultured in 6 well-plates for 48 h prior to downstream experiments [34]. Explants were cultured in DMEM + GlutaMAX™ (1 g/L D-Glucose, +Pyruvate) (21885-025, Life-Technologies) supplemented with 1% (v/v) ITS-X (25030-024, Life-Technologies), 40 µg/mL L-Proline (P5607, Sigma-Aldrich), 1.25 mg/mL Albumax (11020-021, Life-Technologies), 1% (v/v) Penicillin-Streptomycin, 2.5 µg/mL amphotericin B solution and 50 µg/mL L-Ascorbic acid 2-phosphate sesquimagnesium salt hydrate [41]. All tissue and cell culture following extraction and expansion was performed at 5% O_2_, 5% CO_2_ at 37 °C in an O_2_ control in vitro glove box (Coy Laboratory Products, York, UK).

### Metabolic activity of human NP cells following PCRX-201 transduction

NP cells were seeded into 96-well plates at a density of 1 × 10^4^ cells/well. NP cells were treated for 48 h with PCRX-201 at a range of multiplicities of infection (MOI, the number of viral particles per NP cell): 0, 100, 500, 1000, 2000, 3000. Metabolic activity was measured using the resazurin reduction assay as previously described [42]. Metabolic activity after PCRX-201 transduction was performed on NP cells from 3 patients (Table 1a) in triplicate for each MOI.

### Determination of sample size required

Power analysis was completed to determine sample size required to detect a minimum 2 fold difference in output measures for both a Mann-Whitney test and a repeated measures ANOVA were performed (as power analysis for a Kruskal–Wallis was not available). G* Power was utilised to perform a power analysis for the minimum sample size to yield statistical power of at least 80% and an alpha of 0.05. With a minimum sample size determined to be 5 (per group) for Mann–Whitney analysis with actual power calculated as 88%. Whilst G*Power analysis determined a minimum sample size of 6 with actual power of 100% for a repeated measures ANOVA. As such a minimum of 6 donors were utilised for all key outcome measures where greater than 2 groups were tested and a minimum of 5 donors when only 2 groups were investigated. Each donor was then treated in triplicate for each experimental condition investigated.

### Monolayer PCRX-201 transduction and IL-1Ra production

NP cells (*n* = 7 patients in triplicate) (Table 1a) were seeded into Nunc™EasYFlask™ T25 culture flasks (156367, ThermoFisher, Loughborough, UK) at a density of 1 × 10^5^ cells/mL. Cells were transduced with PCRX-201 at MOI: 0, 750 and 3000 for: 2 d ± 1 d 10 ng/mL IL-1β (200-01B, PeproTech, London, UK), or 5 d ± 4 d 10 ng/mL IL-1β. Cell culture supernatant was collected, centrifuged at 600 g for 5 mins and stored at −80 °C prior to analysis. The concentration of IL-1Ra in cell culture supernatant was determined using Human IL-1Ra/IL-1F3 DuoSet ELISA (DY280, R&D Systems, Abingdon, UK) with DuoSet ELISA Ancillary Reagent Kit 2 (DY008B, R&D Systems), following the manufacturer’s protocol. For detecting IL-1Ra with ELISA, cell culture supernatant was used neat.

### 3D cell culture, PCRX-201 transduction and long-term IL-1Ra production

NP cells (*n* = 6 patients in triplicate) (Table 1b) were transduced with PCRX-201 at MOI: 3000, and directly resuspended in sterile-filtered 1.2% w/v alginic acid sodium salt (180947, Sigma-Aldrich) in 0.15 M NaCl, at a density of 4 × 10^6^ cells/mL [43]. Fully gelled alginate beads containing human NP cells were produced as described previously [44]. Alginate beads were cultured in the same media as NP tissue explants [41]. Alginate beads were cultured for up to 10-weeks and media was changed once a week. Cell culture supernatant was retained for analysis 2 d, 1w, 2w, 3w, 4w, 6w, 8w and 10w following transduction with PCRX-201. Non-infected NP cells resuspended in alginate beads served as controls. The concentration of IL-1Ra within cell culture supernatant, across the time course, was determined by ELISA.

### Downstream protein production following 3D PCRX-201 infection and viability

NP cells (*n* = 6 patients in triplicate) (Table 1b) were transduced with PCRX-201 at MOI: 0, 750, or 3000, directly resuspended into alginate beads and cultured for 2w to enable in vivo-like re-differentiation, followed by 1w of culture ±100 pg/mL IL-1β to represent a physiological concentration of IL-1β. Media was changed weekly. Cell culture supernatant was retained for secretome analysis. Following culture, cells were released from alginate via alginate dissolving buffer (55 mM sodium citrate, 30 mM EDTA, 150 mM NaCl) for 10 min at 37 °C, digested in papain (100U papain (P3375, Sigma-Aldrich) in 20 mM sodium phosphate, 1 mM EDTA, 2 mM DTT, pH6.8) at 37 °C overnight with shaking, before total DNA concentration/alginate bead was determined using Quant-iT™ PicoGreen™ dsDNA assay kit (P7589, Life-Technologies) following the manufacturer’s protocol. The secretome within cell culture supernatant was determined using DuoSet ELISAs (all R&D Systems): IL-1Ra (DY280, neat samples), IL-1β (DY201, 1:2 sample dilution), IL-6 (DY206, 1:50 sample dilution), IL-8 (DY208, 1:32 sample dilution), MMP3 (DY513, 1:60 sample dilution), ADAMTS4 (DY4307-05, 1:2 sample dilution), VEGF (DY293B, neat samples). All DuoSet ELISAs were performed using DuoSet ELISA Ancillary Reagent Kit 2 (DY008B, R&D Systems) following the manufacturer’s protocols.

### Paracrine culture of PCRX-201-conditioned media

NP cells (*n* = 6 patients in triplicate) (Table 1b), resuspended in alginate beads and allowed to re-differentiate for 2w, were treated for 3 days with conditioned media harvested from NP cells (in alginate beads) that had been transduced with PCRX-201 (MOI:3000) for 1 week previously. Following culture, the concentration of downstream proteins: IL-1β, IL-6, MMP3, ADAMTS4 and VEGF in cell culture supernatant was determined using ELISA as above. NP cells treated with conditioned media from previously non-infected cells (in alginate beads), from the same patient, were used as controls.

### PCRX-201 explant injection

NP tissue explants (*n* = 5 patients in triplicate) (Table 1c) were injected with PCRX-201 (MOI:3000) in 50 µL PBS. The approximate number of cells within NP explants was determined by calculating the volume of explants (~5 mm^3^ cylindrical shape) and determining the cell number based on the reported cell density of the NP (4 × 10^6^ cells/mL) [43]. PBS-injected NP explants served as controls. Following injection, explants were cultured for 2w, cell culture supernatant harvested, and tissue fixed in 10% NBF (3800600, Leica Biosystems) for 48 h prior to processing into paraffin wax. The concentration of IL-1Ra and downstream proteins, IL-1β, IL-6, MMP3, ADAMTS4, aggrecan (DY1220, R&D Systems) and VEGF released into cell culture supernatant was determined using ELISA. Four-micron sections of paraffin embedded explants were examined using immunohistochemistry (IHC) to determine the cellular expression of IL-1Ra, IL-1β, VEGF, NGF, collagen-type-II, aggrecan, MMP3 and ADAMTS4 following previously published methods [45](Table 1d). Images captured using an Olympus BX60 microscope and CellSens Entry v1.14 software (Olympus, Southend-on-Sea, UK). NP cells within the section, were counted as immunopositive (brown staining) or negative (blue nuclei staining only), using a raster pattern to move across the tissue until a total of 200 cells were counted per sample and percentage immunopositve cells calculated. All immunohistochemical data was performed with the observer blind to treatment groups.

### Statistics

All data were found to be non-parametric, therefore Kruskal–Wallis with Dunn’s multiple comparison tests were performed to determine differences across three or more treatment groups (all monolayer, total time course of long-term IL-1Ra production and 3D downstream protein production experiments). Mann–Whitney tests were used to determine differences between data containing two treatment groups (individual time points for long-term IL-1Ra production, paracrine effects, explant protein production and IHC). All data was included in the analysis. All statistical analysis was performed in Graph Pad Prism v9, and two tailed tests with adjusted *p* values where multiple comparisons were used and accepted as significant if *p* ≤ 0.05.

## Results

### Monolayer viability and IL-1Ra production following PCRX-201 infection

**Fig. 1:**
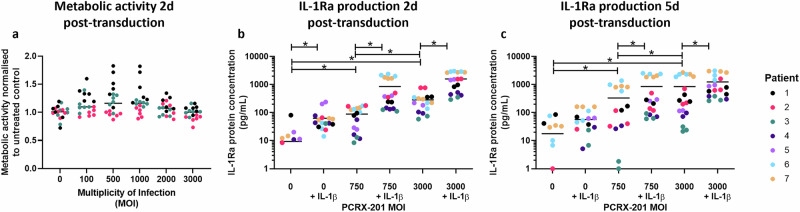
Metabolic activity and IL-1Ra production in monolayer-cultured, PCRX-201-transduced, degenerate human NP cells. **a** Metabolic activity of human NP cells 48 h after transduction with PCRX-201 at different multiplicities of infection (MOI), normalised to untreated controls. IL-1Ra production in cell culture supernatant after (**b**) 2 d PCRX-201 transduction ±1 d 10 ng/mL IL-1β stimulation, **c** 5 d PCRX-201 transduction ±4 d 10 ng/mL IL-1β stimulation. Horizontal lines represent median values. Significant differences determined by Kruskal–Wallis with Dunn’s multiple comparison tests *p ≤ 0.05.

The metabolic activity of human NP cells 48 h after PCRX-201 transduction was not significantly altered across all MOIs investigated (0–3000) (Fig. 1a). IL-1Ra protein production in human NP cells after 48 h PCRX-201 transduction was significantly increased in MOI:750 (*p* = 0.0347) and 3000 (*p* < 0.0001) treatment groups, compared to non-transduced controls (Fig. 1b). IL-1Ra production was also significantly increased following IL-1β stimulation, in non-transduced controls+IL-1β (*p* = 0.0389), and those transduced with MOI:750 + IL-1β for 48 h compared to MOI:750 (*p* < 0.0001) (Fig. 1b), and MOI:3000 + IL-1β for 48 h compared to MOI:3000 (*p* < 0.0001) (Fig. 1b). A significant increase in IL-1Ra was also seen between MOI:750 and 3000 without IL-1β stimulation (*p* < 0.0001) (Fig. 1b). After 5 d PCRX-201 transduction, MOI:750 (*p* = 0.0321) and MOI:3000 (*p* < 0.0001) treatments significantly increased IL-1Ra compared to non-transduced controls (Fig. 1c). IL-1Ra production was significantly increased in both MOI:750 + IL-1β (*p* = 0.0315) and MOI:3000 (*p* = 0.0472) compared to MOI:750 (Fig. 1c). Production of IL-1Ra was significantly increased after MOI:3000 + IL-1β compared to MOI:3000 (*p* = 0.0474) (Fig. 1c).

### Long-term IL-1Ra release

**Fig. 2:**
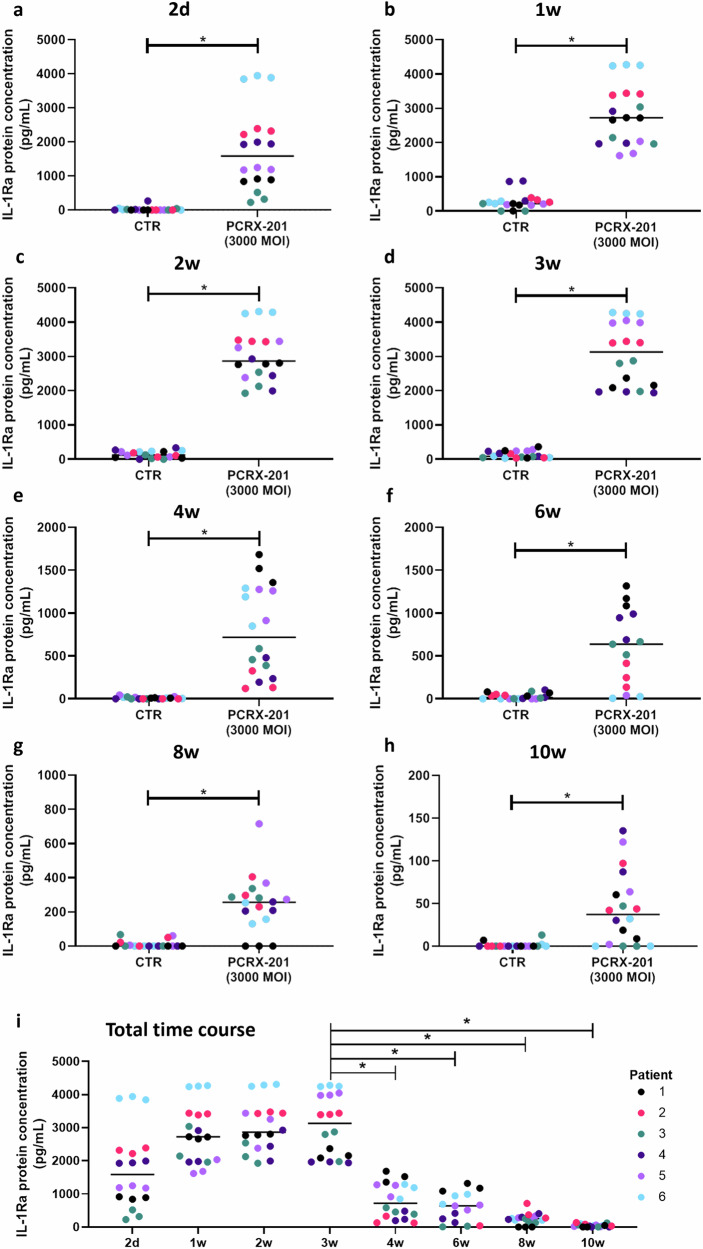
Long-term production of IL-Ra following initial PCRX-201 transduction in 3D alginate bead-cultured human NP cells. IL-1Ra release into cell culture supernatant (**a**) 2 d, (**b**) 1w, (**c**) 2w, (**d**) 3w, (**e**) 4w, (**f**) 6w, (**g**) 8w and (**h**) 10w after initial transduction with PCRX-201 at a multiplicity of infection (MOI) of 3000, compared to non-infected control cells (CTR). **i** Time course of IL-1Ra release from 2 d to 10w in PCRX-201 transduced human NP cells (MOI:3000). Horizontal lines represent median values. Significant differences determined by (**a**–**h**) Mann–Whitney or (**i**) Kruskal–Wallis with Dunn’s multiple comparison tests *p ≤ 0.05.

Production of IL-1Ra in cell culture supernatant from 3D alginate bead-cultured human NP cells was significantly increased following PCRX-201 transduction, after all time points (all *p* < 0.0001), compared to non-transduced controls at each timepoint (Fig. 2a–h). Production of IL-1Ra was significantly decreased after 4w (*p* = 0.0002), 6w (*p* < 0.0001), 8w (*p* < 0.0001) and 10w (*p* < 0.0001) compared to 3w, post transduction (Fig. 2i), however, a decrease in the integrity of alginate beads was observed after 6w in culture, indicating effects may also be due to a loss of cells from beads.

### Downstream protein production following PCRX-201 infection

**Fig. 3:**
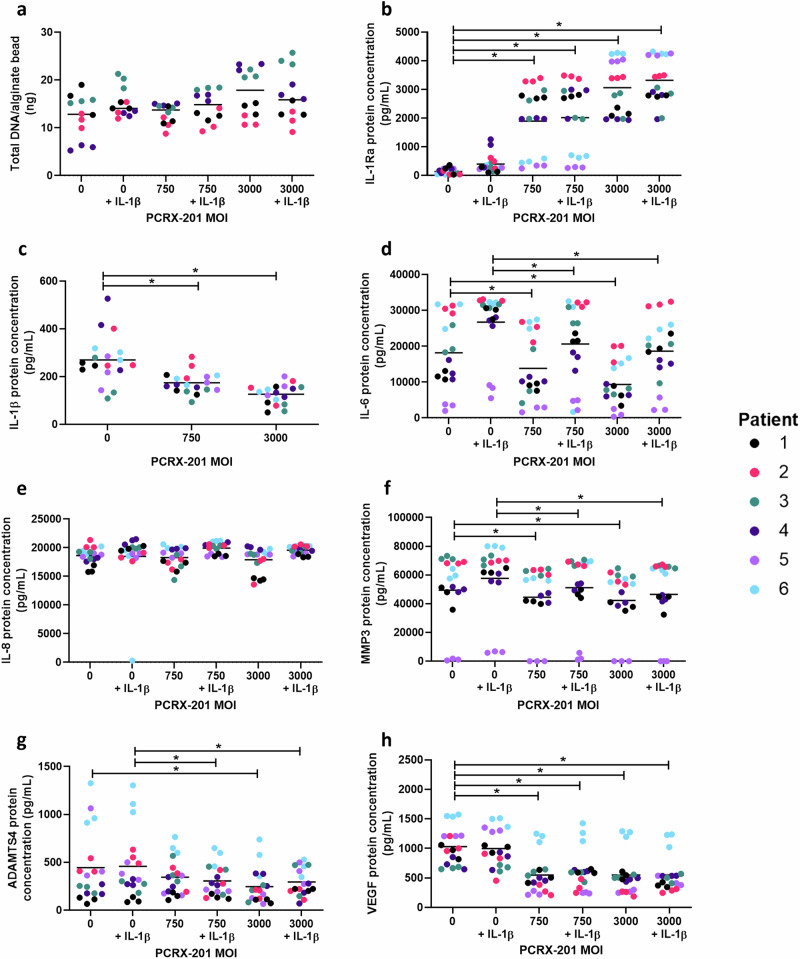
Viability and downstream protein production in 3D cultured human NP cells following PCRX-201 transduction. Degenerate human NP cells (*n* = 6 patients in triplicate) were transduced with PCRX-201 at a multiplicity of infection (MOI) of 0, 750 or 3000, resuspended in alginate beads and cultured for 3 weeks, +/−100 pg/mL IL-1β for the last week of culture. Following culture (**a**) total DNA/alginate bead was measured to determine effects of culture on NP cell viability. ELISA was used to determine the downstream production of (**b**) IL-1Ra, (**c**) IL-1β, (**d**) IL-6, (**e**) IL-8, (**f**) MMP3, (**g**) ADAMTS4, and (**h**) VEGF within cell culture supernatant after 3w culture. Horizontal lines represent median values. Significant differences determined by Kruskal–Wallis with Dunn’s multiple comparison tests *p ≤ 0.05.

Following initial PCRX-201 transduction, and 3w alginate bead culture, there were no significant differences in total DNA/alginate bead content across any PCRX-201 MOI (+/-IL-1β) (Fig. 3a). IL-1Ra protein concentration was significantly increased in MOI:750, MOI:750 + IL-1β, MOI:3000 and MOI:3000 + IL-1β (all *p* < 0.0001) compared to non-transduced controls (Fig. 3b). The production of IL-1β in MOI:750 (*p* = 0.0169) and MOI:3000 (*p* < 0.0001) was significantly decreased compared to non-transduced controls (Fig. 3c). The production of IL-6 in MOI:750 (*p* = 0.0438) and MOI:3000 (*p* = 0.0290) was significantly decreased compared to non-transduced controls (Fig. 3d) and decreased in MOI:750 + IL-1β (*p* = 0.0418) and MOI:3000 + IL-1β (*p* = 0.0300) compared to non-transduced +IL-1β controls (Fig. 3d). There were no significant differences in the production of IL-8 across all treatment groups (Fig. 3e). The production of MMP3 in MOI:750 (*p* = 0.0431) and MOI:3000 (*p* = 0.0422) was significantly decreased compared to non-transduced controls (Fig. 3f) and decreased in MOI:750 + IL-1β (*p* = 0.0475) and MOI:3000 + IL-1β (*p* = 0.0066) compared to non-transduced+IL-1β controls (Fig. 3f). Of note one sample (Patient 5) showed low expression of IL-6 and MMP 3 across all treatment groups, this patient was the only sample from a non-degenerate disc (Grade 2-Table 1b). The production of ADAMTS4 was significantly reduced in MOI:3000 (*p* < 0.0001) compared to non-transduced controls and reduced in MOI:750 + IL-1β (*p* = 0.0034) and MOI:3000 + IL-1β (*p* = 0.0015) compared to non-transduced+IL-1β controls (Fig. 3g). VEGF protein concentration was significantly decreased in MOI:750 (*p* = 0.0014), MOI:750 + IL-1β (*p* = 0.0073), MOI:3000 (*p* = 0.0008) and MOI:3000 + IL-1β (*p* = 0.0097) compared to non-transduced controls (Fig. 3h).

### Paracrine effects of PCRX-201 conditioned media

**Fig. 4:**
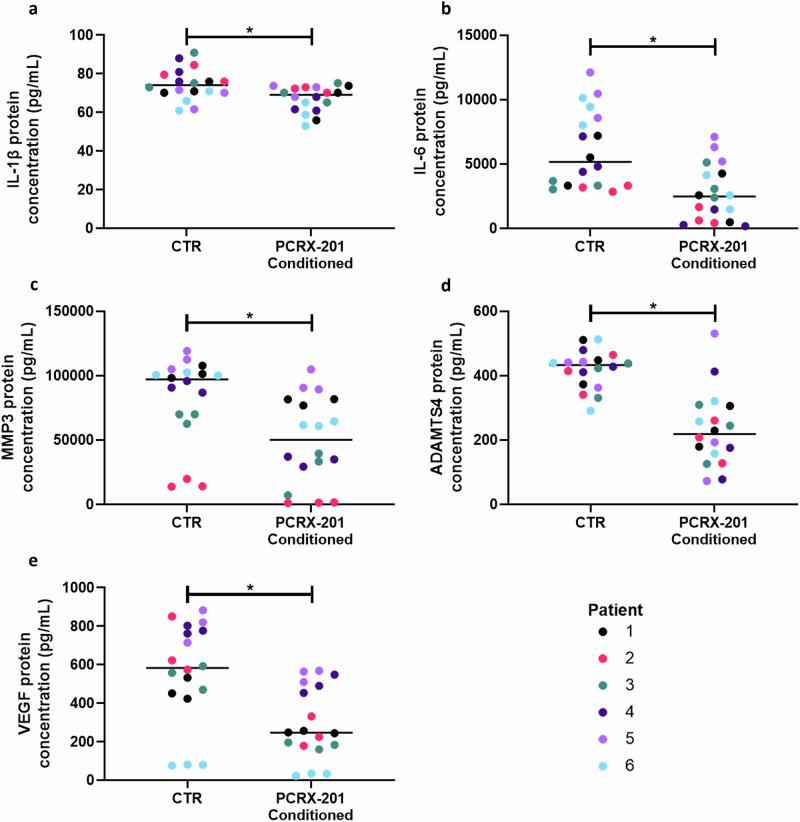
Paracrine effect on downstream protein production in 3D alginate bead-cultured human NP cells when exposed to PCRX-201-conditioned media. Degenerate human NP cells (*n* = 6 patients in triplicate) encapsulated in alginate beads were treated for 3 days with conditioned media from NP cells transduced with PCRX-201 (MOI:3000) for 1 week previously. Cells treated with media from previously non-transduced cells served as controls (CTR). ELISA was used to determine the effects on downstream production of (**a**) IL-1β, (**b**) IL-6, (**c**) MMP3, (**d**) ADAMTS4 and (**e**) VEGF within cell culture supernatant after 3 days of culture. Horizontal lines represent median values. Significant differences determined by Mann-Whitney tests *p ≤ 0.05.

The protein concentration of IL-1β (*p* = 0.0058), IL-6 (*p* = 0.0004), MMP3 (*p* = 0.0032), ADAMTS4 (*p* < 0.0001) and VEGF (*p* = 0.0021) was significantly decreased in NP cells treated with PCRX-201 conditioned media compared to NP cells treated with control media (Fig. 4a–e, respectively).

### Explant secreted protein production following PCRX-201 injection

**Fig. 5:**
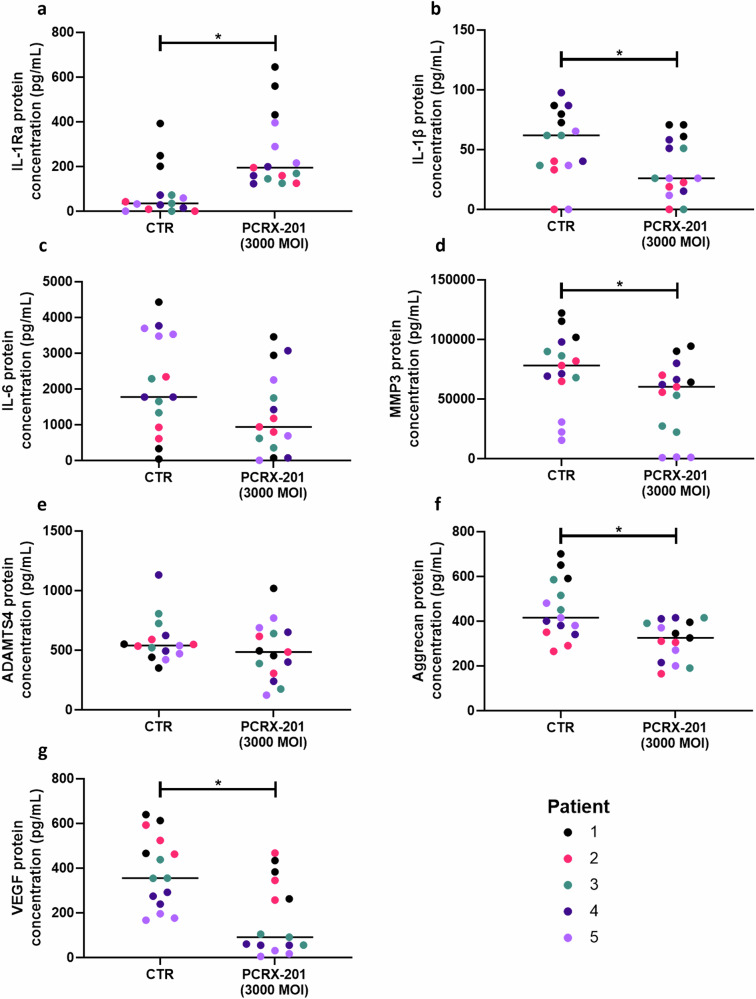
Modulation of IL-1Ra and downstream protein production in ex vivo human NP tissue explants following direct PCRX-201 injection. Human NP explants (*n* = 5) (Table 1) were injected with PCRX-201 at a multiplicity of infection (MOI) of 3000 and cultured for 2 weeks within a semi-constrained plastic ring to limit tissue swelling. Non-transduced tissue explants served as controls (CTR). Following culture, the production of (**a**) IL-1Ra, (**b**) IL-1β, (**c**) IL-6, (**d**) MMP3, (**e**) ADAMTS4, (**f**) aggrecan, and (**g**) VEGF within cell culture supernatant was determined using ELISA. Horizontal lines represent median values. Significant differences determined by Mann–Whitney tests *p ≤ 0.05.

The production of IL-1Ra in human ex vivo NP explants following PCRX-201 injection was significantly increased compared to non-injected control explants (*p* = 0.0003) (Fig. 5a). The production of IL-1β (*p* = 0.0437), MMP3 (*p* = 0.0235), aggrecan (*p* = 0.0063) and VEGF (*p* = 0.0023) was significantly decreased in PCRX-201-injected explants compared to controls (Fig. 5b, d, f and g, respectively). The production of IL-6 and ADAMTS4 were not significantly altered in PCRX-201 injected explants compared to controls (Fig. 5f, h).

### Native explant protein expression following PCRX-201 injection

**Fig. 6:**
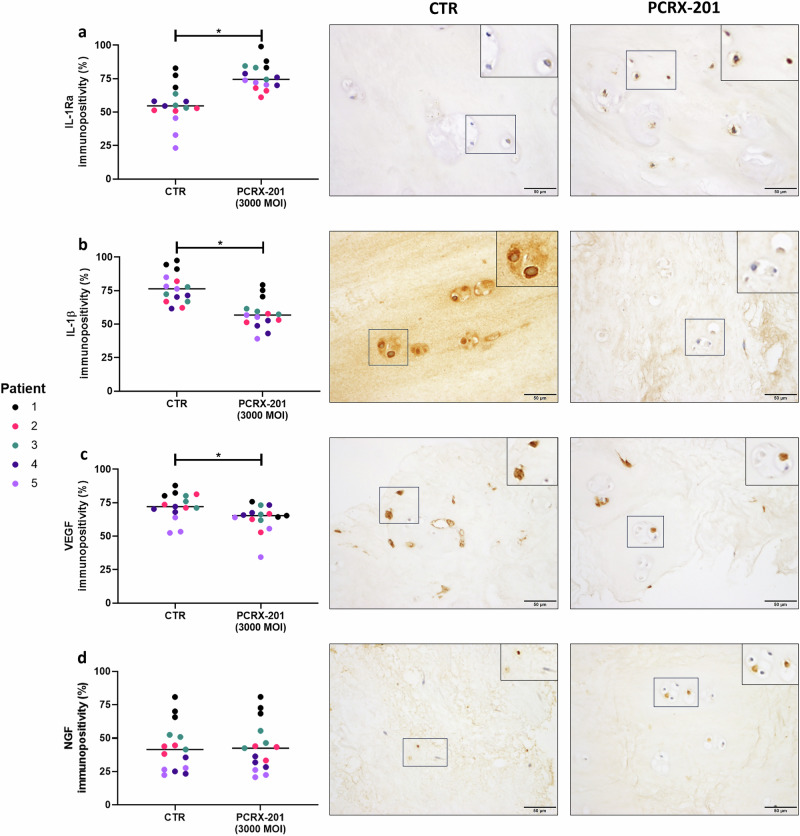
Expression of IL-1Ra and catabolic proteins in ex vivo human NP tissue explants following direct PCRX-201 injection. Expression of (**a**) IL-1Ra, (**b**) IL-1β, (**c**) VEGF and (**d**) NGF in native human nucleus pulposus (NP) cells, determined by immunohistochemistry, following 2-week ex vivo culture of human NP tissue explants (*n* = 5). Immunopositivity (brown staining) was determined by counting 200 NP cells per sample and expressing the percentage of cells positively stained for proteins of interest. Cell nuclei counterstained blue with Mayer’s haematoxylin. CTR non-transduced control tissue, PCRX-201 tissue transduced with PCRX-201 at a multiplicity of infection (MOI) of 3000. Horizontal lines represent median values. Significant differences determined by Mann–Whitney tests *p ≤ 0.05.

**Fig. 7:**
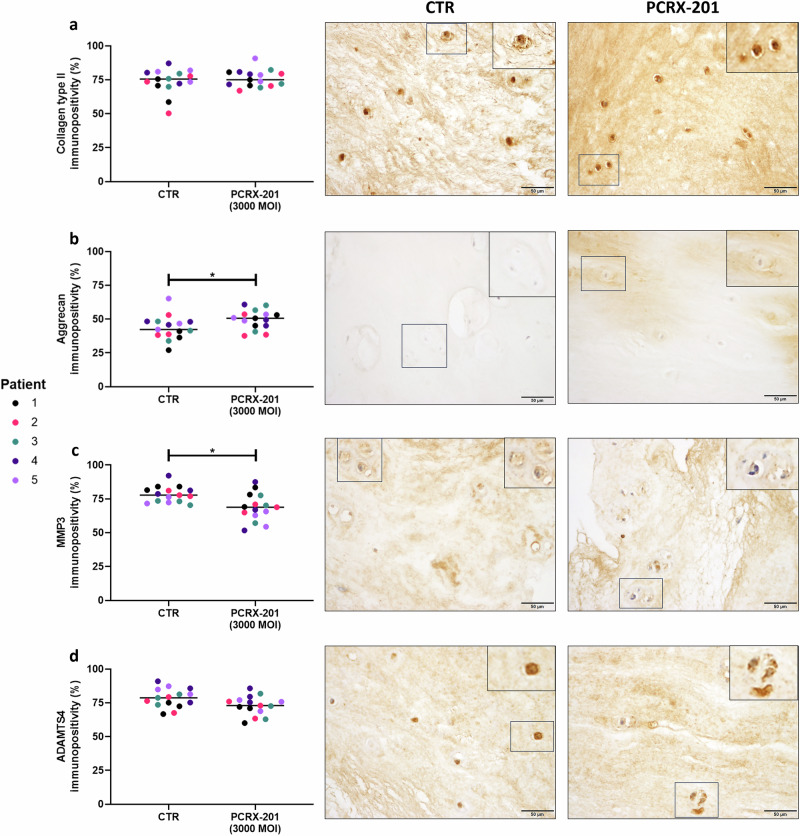
Expression of extracellular matrix proteins and matrix degrading enzymes in ex vivo human NP tissue explants following direct PCRX-201 injection. Expression of (**a**) collagen type II, (**b**) aggrecan, (**c**) MMP3 and (**d**) ADAMTS4 in native human nucleus pulposus (NP) cells, determined by immunohistochemistry, following 2-week ex vivo culture of human NP tissue explants (*n* = 5). Immunopositivity (brown staining) was determined by counting 200 NP cells per sample and expressing the percentage of cells positively stained for proteins of interest. Cell nuclei counterstained blue with Mayer’s haematoxylin. CTR non-transduced control tissue, PCRX-201 tissue transduced with PCRX-201 at a multiplicity of infection (MOI) of 3000. Horizontal lines represent median values. Significant differences determined by Mann–Whitney tests *p ≤ 0.05.

The percentage of native NP cells with immunopositive staining for IL-1Ra significantly increased in NP explants following PCRX-201-injection and 2 weeks culture, compared to non-injected controls (*p* < 0.0001) (Fig. 6a). The percentage of cells with immunopositive staining for IL-1β (*p* < 0.0001) and VEGF (*p* = 0.0128) were significantly reduced in PCRX-201-injected explants compared to controls (Fig. 6b, c). The percentage of cells with immunopositive staining for NGF (Fig. 6d), collagen-type-2 (Fig. 7a) and ADAMTS4 (Fig. 7d) did not change across treatment groups. The percentage of cells with immunopositive staining for aggrecan was significantly increased in PCRX-201-injected explants compared to controls (*p* = 0.0367) (Fig. 7b). The percentage of cells with immunopositive staining for MMP3 (*p* = 0.0027) was significantly reduced in PCRX-201-injected explants compared to controls (Fig. 7d).

## Discussion

The IVD is a finely balanced tissue, where native cells have adapted to a challenging environment to produce and turnover ECM in-tune with the requirements of the disc. When this balance is lost, IVD degeneration and the onset of LBP can occur. IL-1 has a major role in disc homeostasis; expression of IL-1 is vital for healthy ageing of the IVD [46]. Yet, over-expression, which is not matched by IL-1Ra, results in a catabolic shift leading to tissue destruction and IVD degeneration [9, 47]. Highlighting that reducing IL-1 signalling may be a promising target for inhibiting ECM destruction and alleviating the symptoms of LBP. This study, for the first time investigated the efficacy of PCRX-201 to transduce human IVD cells and tissue isolated from degenerate IVDs, displaying increased production of IL-1Ra protein, and reduced production of downstream catabolic factors associated with IVD degeneration.

The modulation of anti-catabolic proteins has been proposed as a target for a gene therapy approach to IVD degeneration. A number of proteins including: transforming growth factor beta 1 (TGF-β1); bone morphogenic protein-2 (BMP-2); BMP-12 and tissue inhibitor of metalloproteinase 1 (TIMP1); have been successfully up-regulated within IVDs following viral vector delivery [48–51]. These factors have demonstrated inhibition of degenerative changes in transduced IVDs [48–51]. Highlighting the sustained production of therapeutic agents for the treatment of IVD degeneration can be achieved by the application of gene therapy. Previous investigations into IL-1Ra gene therapy within human IVD cells showed that only cells derived from non-degenerate NP tissue were able to respond with regards to increased IL-1Ra protein production [34, 35]. Whereas here, PCRX-201 was able to transduce and increase the production of IL-1Ra in human NP cells derived from degenerate IVDs, with no adverse effects on cell viability. This is beneficial as increased IL-1 expression during degeneration becomes unrestrained, as IL-1Ra does not follow the same trend [24]. It is unclear why PCRX-201 was able to induce IL-1Ra protein production in cells derived from degenerate disc tissue, whilst the previous adenoviral vector system was only successful in cells derived from non-degenerate disc tissue [34, 35]. The differential effects seen could have been due to the previous studies utilising an earlier generation of adenoviral vector which retained more viral genome which could have inhibited its successful transduction into the degenerate disc cells. Furthermore, the current PCRX-201 vector utilises an inducible promotor (NFkB), whilst the previous vector deployed the constitutive CMV promotor. Given that cells from degenerate discs are likely to have high activation of NFkB, this could have led to improved IL-1Ra production.

By introducing PCRX-201, IL-1Ra levels can be increased, dampening the effects of IL-1. Initial studies to test PCRX-201 infection were performed on NP cells grown in monolayer, which is far from being physiologically relevant and causes NP cells to de-differentiate [52, 53]. Three-dimensional alginate bead culture has been shown to enable the re-differentiation of isolated IVD cells in comparison with monolayer culture, restoring extracellular matrix expression and cellular metabolism, indicating it is a more physiologically-relevant in vitro culture system [52, 53]. Therefore, this culture method was utilised here to more accurately determine long-term IL-1Ra release, downstream protein production and paracrine effects of PCRX-201 on human IVD cells. Furthermore, cell and tissue explant culture were performed under 5% O_2_, low glucose and a serum free culture environment. These conditions mimic the in vivo environment of the IVD [7] whilst previous studies investigating IL-1Ra gene therapy and release, were performed under 21% O_2_, high glucose, and in the presence of FCS [32, 34, 35], which could have also contributed to differential findings observed.

Increased levels of IL-1Ra were observed from PCRX-201-transduced cells for up to 10 weeks following initial transduction, indicating PCRX-201 enables degenerate cells to durably produce elevated levels of IL-1Ra. However, one caveat is that following 6 weeks of culture, the integrity of alginate beads decreased, which may have resulted in the loss of cells from culture and thus under-represented the levels of IL-1Ra produced and could partly explain the reduction seen in longer term alginate cultures. For future studies, the use of larger alginate constructs may provide more accurate measurements of IL-1Ra release, providing greater structural integrity. Furthermore, the use of IVD tissue explants for long term studies of PCRX-201 transduction would be representative of in vivo effects. These longer-term cultures particularly using tissue explants would also enable investigation of potential actions of improving cellularity of the disc combating effects of apoptosis and senescence which lead to decreased cellularity within disc degeneration [14–17]. Due to the short time frames investigated within the current study these were not investigated outcome measures.

IL-1Ra release from alginate beads was greatest following 3w. Therefore, this time point was chosen to investigate the effects of PCRX-201-driven IL-1Ra production on the release of downstream catabolic factors. Three weeks of culture also enabled the optimal time for re-differentiation of alginate-encapsulated NP cells, 2w [52], plus an additional 1w of 100 pg/mL IL-1β stimulation. The significant reduction in the production of IL-1β, IL-6, MMP3, ADAMTS4 and VEGF indicates that the levels of IL-1Ra produced were able to inhibit IL-1 signalling and its downstream effects. Although the anti-catabolic actions of PCRX-201 correlated with the increased level of IL-1Ra induced, it is possible that some of the anti-IL-1 actions were due to the use of the NFkB response elements to induce IL-1Ra, which could have led to competition from viral genomes for the NFkB response elements. However, given the increased production of IL-1Ra seen following PCRX-201 it is likely most of the anti-catabolic actions were due to the increased IL-1Ra. Although the utilisation of a control vector also under NFkB regulation would address this possibility, such a vector was not available and given the ultimate outcome of the PCRX-201 is IL-1 inhibition, which was observed, the addition of such a control vector was not deemed necessary.

This highlights that PCRX-201 can potentially reverse the catabolic cellular changes that IL-1 induces during IVD degeneration, inhibiting tissue destruction [24, 25]. Whereas, in previous studies gene therapy only enabled an increase in IL-1Ra in non-degenerate NP cells and a decrease in expression of MMPs [34, 35]. IL-8 was produced in very high levels across all treatment groups which may have caused saturation within cell culture supernatant, leading to a loss in detection of any differences in IL-8 concentration. Stimulation of NP cells with IL-1β did not significantly increase the production of any downstream catabolic factors associated with degeneration (a trend was observed for IL-6 but did not reach significance). As NP cells were extracted from degenerate tissues, levels of catabolic protein expression may have already been induced by elevated levels of IL-1 in vivo. Furthermore, cells were treated with 100 pg/mL IL-1β, however non-transduced, 3D-cultured NP cells produced higher levels of IL-1β in culture (108–526 pg/mL, median: 253 pg/mL). This indicates the treatment level would not alter catabolic protein production substantially, as NP cells were already secreting IL-1β at levels higher than the treatment supplemented into cultures.

PCRX-201-driven production of IL-1Ra also decreased catabolic protein release in a paracrine manner. This indicates the levels of IL-1Ra produced by PCRX-201-transduced NP cells is increased sufficiently to elicit an anti-degenerative response in cells that have not previously been transduced, signifying the therapeutic potential of PCRX-201. IL-1Ra collected from PLGA microspheres decreased gene expression of catabolic mediators when NP cells were treated in a similar manner [32]. However, this study utilised healthy bovine NP cells stimulated with supraphysiological levels of IL-1β (10 ng/mL), to induce catabolic gene expression [32]. This makes comparisons between this study difficult as healthy bovine NP cells treated with IL-1β may not accurately represent degenerate human NP cells and the paracrine effects observed in the current study.

Direct injection of PCRX-201 into NP tissue explants is physiologically relevant and anti-catabolic effects were observed after only 2w following injection, regarding a reduction in the release of catabolic proteins into cell culture supernatant and their expression within native NP cells. Not all catabolic proteins were significantly reduced, but trends were observed (IL-6, ADAMTS4), suggesting longer-term culture of PCRX-201-injected explants may provide improved anti-catabolic effects. Aggrecan released from PCRX-201-injected explants into cell culture supernatant was reduced, but native cell expression, determined by IHC, was increased. This demonstrates native NP cells increase synthesis and expression of aggrecan which remains within and forms part of the ECM, whereas, aggrecan degradation and the release of fragments into cell culture supernatant is reduced [54], due to a decrease in catabolic enzyme expression and production.

All cultures were performed under conditions which closely mimic the physiology of the disc providing strong evidence that PCRX-201 would have the ability to infect IVD cells in vivo, however not all conditions were mimicked. Diurnal biomechanical loading and hydrostatic pressure have significant impacts of disc health and cellular function [6–8]; the diffusion and transduction of PCRX-201 within IVD tissue was not investigated under these important physiological cues. Future work including direct injection of PCRX-201 into ex vivo human NP explants subjected to diurnal physiological loading (amongst all other physiological conditions) would be helpful to gain further understanding into the efficacy of PCRX-201 to elicit anti-catabolic effects within the IVD.

PCRX-201 is a promising IL-1Ra gene therapy that has shown potential for the treatment of OA [37]. Here, for the first time we show that PCRX-201 can transduce degenerate IVD cells, decrease production of catabolic proteins responsible for driving IVD degeneration and induce the expression of the important matrix component aggrecan. Results indicate that PCRX-201 may provide disease-modifying effects to the cells of the degenerate IVD and supports continued development of PCRX-201 into a viable therapy aimed at targeting IVD degeneration and LBP.

## Data Availability

All data generated within this study can be found within the published figures, access to raw data files can be made on request to the corresponding author on reasonable request.
